# Beating the ER: novel insights into FAM134B function and regulation

**DOI:** 10.15252/embj.2020104546

**Published:** 2020-02-19

**Authors:** Chiara De Leonibus, Laura Cinque, Carmine Settembre

**Affiliations:** ^1^ Telethon Institute of Genetics and Medicine (TIGEM) Pozzuoli Italy; ^2^ Department of Medical and Translational Science University of Naples “Federico II” Naples Italy

**Keywords:** Autophagy & Cell Death, Post-translational Modifications, Proteolysis & Proteomics

## Abstract

To maintain cellular homeostasis, the endoplasmic reticulum (ER) necessitates a continuous removal of ER fragments via a selective, receptor‐mediated, form of autophagy known as ER‐phagy. In this issue of *The EMBO Journal*, Jiang *et al* (2020) shed light on how the best characterized autophagy receptor FAM134B mediates ER membrane fragmentation, the earliest event during ER‐phagy. They propose a dynamic model for FAM134B protein oligomerization and ER membrane scission, which are driven by CAMK2B‐mediated phosphorylation of the receptor and are altered in sensory neuropathy.

## Graphical abstract

A new study reveals that oligomerization of the autophagy receptor FAM134B drives endoplasmic reticulum fragmentation and ER‐phagy, two processes deregulated in hereditary neuropathies.

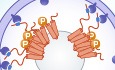

The endoplasmic reticulum (ER) is the largest cellular organelle, forming a continuous membrane network of cisternae and tubules spreading from the nucleus to the plasma membrane (Schwarz & Blower, 2016). The ER plays fundamental cellular functions, including calcium storage, protein and lipid biosynthesis (Schwarz & Blower, 2016). In order to maintain cellular homeostasis, the ER adapts its shape and size in response to cellular stress conditions (Walter & Ron, 2011). In mammals, “ER‐phagy” is as a selective form of ER degradation through the engulfment of ER fragments into autophagosomes for lysosomal delivery (Khaminets *et al*, 2015; De Leonibus *et al*, 2019). This process needs ER‐phagy receptors, which are ER‐resident proteins that harbour a LC3 interaction motif (LIR) enabling the binding of the cargo to LC3/GABARAP family proteins on autophagosomal membranes (Stolz *et al*, 2014). FAM134B was the first and so far the best characterized ER‐phagy receptor (Khaminets *et al*, 2015). FAM134B is an intramembrane ER‐resident protein that contains a LIR domain and a reticulon homology domain (RHD), which consists of two membrane‐embedded hydrophobic regions bridged by a flexible cytoplasmic linker (Khaminets *et al*, 2015; Bhaskara *et al*, 2019). In addition to ER turnover, FAM134B‐mediated ER‐phagy exerts ER quality control functions by facilitating the degradation of inappropriately folded ER clients (Cui *et al*, 2019; Forrester *et al*, 2019).

What is currently missing in the field of ER‐phagy, and specifically in selective autophagy, is the understanding of the intracellular signals that govern these types of processes. Jiang *et al* describe a series of molecular events that trigger FAM134B‐mediated ER‐phagy. First, they demonstrated that the RHD of FAM134B drives receptor oligomerization and that this event promotes ER fragmentation prior to LIR‐mediated ER‐phagy. Similar findings have been recently reported by another group using molecular modelling and dynamics simulations (Bhaskara *et al*, 2019). Second, the authors identified three potential phosphorylation sites [serine149, serine151 and serine153 (S149, S151 and S153)] within the flexible cytoplasmic linker that bridges the two intermembrane hairpins of the RHD. Using site‐directed mutagenesis, they demonstrated that the serine residues S149, S151 and S153 regulate FAM134B oligomerization and, in turn, ER fragmentation during ER‐phagy. Third, Jiang and co‐workers identified the Calcium/Calmodulin Dependent Protein Kinase II Beta (CAMK2B) as a putative kinase responsible for the phosphorylation of FAM134B at S151. Consistently, CAMK2B activators (ionomycin and EB1089) or inhibitors (KN‐93 or CAM2KB‐specific knockdown), respectively, stimulated or repressed ER fragmentation and ER‐phagy in a FAM134B‐dependent manner. Taken together, these data demonstrate the existence of specific signalling events that induce ER‐phagy by favouring the oligomerization of FAM134B.

Loss‐of‐function mutations in FAM134B were identified to be responsible for the pathogenesis of a hereditary sensory and autonomic neuropathy (HSAN‐II) (Kurth *et al*, 2009). Jiang *et al* studied a disease‐associated FAM134B missense variant (FAM134B^G216R^) in the RHD with unknown pathogenic significance (Davidson *et al*, 2012). Surprisingly, FAM134B^G216R^ exhibited gain‐of‐function activity by inducing FAM134B oligomerization, ER scission and ER‐phagy more efficiently than the wild‐type form. Notably, they demonstrated that FAM134B^G216R^ expression affected the survival of dorsal root ganglion (DRG) sensory neurons. Together, these data suggest that FAM134B^G216R^ acts as a gain‐of‐function mutation that might trigger sensory neuronal cell death by inducing excessive ER fragmentation and ER‐phagy (Fig 1). Interestingly, chemical inhibition of CAMK2B activity partly rescued the cytotoxicity in DRG neurons infected with FAM134B^G216R^ variant, suggesting that targeting FAM134B oligomerization might be beneficial for a subset of patients affected by HSAN‐II.

**Figure 1:**
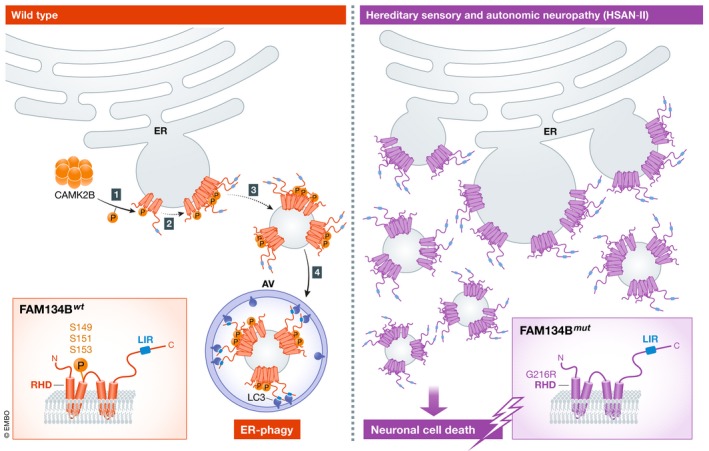
Proposed model of FAM134B oligomerization and regulation CAMK2B‐mediated phosphorylation of FAM134B (1) enhances its self‐association and oligomerization (2), thus promoting ER membrane fragmentation (3) and the ER‐phagy process (4). FAM134B oligomerization can be pathologically enhanced in type II HSAN syndrome due to G216R mutation in the RHD of FAM134B. As a consequence, an excessive ER fragmentation and ER‐phagy may promote neuronal cell death through unknown mechanisms.

In summary, this work offers novel insights into the understanding of FAM134B function. It further opens new avenues on intracellular signals that govern selective forms of autophagy and on the dysfunctional cellular mechanisms beneath the pathogenesis of sensory neuropathy.
